# Barriers to cervical cancer screening among ethnic minority women: a qualitative study

**DOI:** 10.1136/jfprhc-2014-101082

**Published:** 2015-01-12

**Authors:** Laura A V Marlow, Jo Waller, Jane Wardle

**Affiliations:** 1 Senior Research Associate, Cancer Research UK Health Behaviour Research Centre, Department of Epidemiology and Public Health, University College London, London, UK; 2 Principal Research Associate, Cancer Research UK Health Behaviour Research Centre, Department of Epidemiology and Public Health, University College London, London, UK; 3 Professor of Clinical Psychology, Cancer Research UK Health Behaviour Research Centre, Department of Epidemiology and Public Health, University College London, London, UK

**Keywords:** cervical screening, ethnic minority and cultural issues, qualitative research

## Abstract

**Background:**

Ethnic minority women are less likely to attend cervical screening.

**Aim:**

To explore self-perceived barriers to cervical screening attendance among ethnic minority women compared to white British women.

**Design:**

Qualitative interview study.

**Setting:**

Community groups in ethnically diverse London boroughs.

**Methods:**

Interviews were carried out with 43 women from a range of ethnic minority backgrounds (Indian, Pakistani, Bangladeshi, Caribbean, African, Black British, Black other, White other) and 11 White British women. Interviews were recorded, transcribed verbatim and analysed using Framework analysis.

**Results:**

Fifteen women had delayed screening/had never been screened. Ethnic minority women felt that there was a lack of awareness about cervical cancer in their community, and several did not recognise the terms ‘cervical screening’ or ‘smear test’. Barriers to cervical screening raised by all women were emotional (fear, embarrassment, shame), practical (lack of time) and cognitive (low perceived risk, absence of symptoms). Emotional barriers seemed to be more prominent among Asian women. Low perceived risk of cervical cancer was influenced by beliefs about having sex outside of marriage and some women felt a diagnosis of cervical cancer might be considered shameful. Negative experiences were well remembered by all women and could be a barrier to repeat attendance.

**Conclusions:**

Emotional barriers (fear, embarrassment and anticipated shame) and low perceived risk might contribute to explaining lower cervical screening coverage for some ethnic groups. Interventions to improve knowledge and understanding of cervical cancer are needed in ethnic minority communities, and investment in training for health professionals may improve experiences and encourage repeat attendance for all women.

**Key message points**

- Emotional barriers may contribute to lower cervical screening rates among Asian women.
- Interactions with the health professional taking the sample are well remembered and may influence future behaviour.
- Clarification of what cervical screening is should be included in information for ethnic minority women.

## Introduction

In England, cervical screening is recommended for all women aged 25–64 years.^1^ Overall coverage is good, although around one-fifth of eligible women have not been screened within the last 5 years.^1^ These women tend to be younger, unmarried and have fewer educational qualifications.^2^ ^3^ In addition, ethnicity has been associated with lower attendance for cervical screening,^2^ ^4^ ^5^ even after adjusting for socioeconomic status.^2^ This association between ethnicity and screening attendance is concerning, and despite policy commitment to addressing this inequality (i.e. The Cancer Reform Strategy^6^), research exploring the reasons behind it is minimal.

Perceived barriers to cervical screening in the context of an organised programme have been found to include dislike of the test, not wishing to know the result, and the belief that screening is unnecessary in the absence of symptoms.^7–9^ Other priorities, mistrust in the health service, and inconvenient appointment times may also be important.^7–9^ A few studies have explored barriers to cervical screening among specific population subgroups, sampling on the basis of ethnicity, language spoken, migration status or religion. For example, a recent study of attitudes to cervical cancer screening among Somali women in London^10^ found lack of knowledge about the purpose of screening, language difficulties, embarrassment or fear of the test, negative past experiences and practical difficulties were all presented as barriers. An older study that recruited non-English-speaking women in 1994 found the main barriers to cervical screening to be administrative (not receiving an invitation), language-related (a written English invitation and no access to bilingual advocates) and concerns about the location (i.e. sterility, inadequate premises).^11^ More recently, a study of Eastern European migrants also suggested language to be a barrier, alongside lack of awareness, time pressures and negative attitudes to the National Health Service (NHS).^12^ Two studies primarily designed to explore attitudes to human papillomavirus (HPV) testing among Hindu and Muslim women suggested similar barriers.^13^ ^14^ A study of immigrant women in Sweden suggested that women felt their health was not considered a priority in their home countries and as a result they had positive attitudes towards the availability of health care in Sweden, but understanding invitations and making appointments was difficult.^15^

Around 3 million ethnic minority women in England are eligible for cervical screening (i.e. are aged 25–64 years). [NB. Based on 2011 Census data on ethnic group by sex that shows 5 434 151 females are from an ethnic group other than White British and on ethnic group by age that shows 55% of ethnic minorities are aged 25–64 years. http://www.nomisweb.co.uk/census/2011/lc2101ew.] The present study aimed to explore barriers to cervical screening among ethnic minority women living in London and compare these barriers to those raised by women from White British backgrounds.

## Methods

For the purpose of this study, ethnicity was self-defined by participants using the 2001 Census question on ethnic group. The Census is a mandatory survey completed by all households in Great Britain every 10 years. The 2001 ethnicity question asked “What is your ethnic group?”, offering 16 options under five major headings: White; Mixed; Asian or Asian British; Black or Black British; Chinese or other ethnic group^16^ (see reference for the full question). A woman was considered to be from an ethnic minority background if she selected any category other than “White (English/Welsh/Scottish/Northern Irish/British)”.

### Participants

Women aged 25–64 years were recruited through community groups in seven London boroughs with ethnically diverse populations (Brent, Barnet, Hounslow, Hillingdon, Newham, Lewisham and Camden). Online lists of community groups for each borough were obtained through council websites and groups were contacted by email or telephone and asked to advertise the study. Women were either approached directly by the group leader and invited to take part, or responded to the researcher after listening to a verbal description of the study or seeing a poster at the community group venue. An advert about the study was also placed on one council website. We interviewed all women who expressed an interest in taking part.

### Procedure

Interviews took place between June and October 2011. Most interviews were carried out by the first author in English, but 13 were carried out by ethnically matched interviewers. Women were given the option to be interviewed in a language other than English. Interviews lasted approximately 40 minutes, were recorded and were transcribed verbatim. When interviews were not in English (*n*=5) they were transcribed into English and checked by another researcher. The study was approved by the University College London Research Ethics Committee.

### Materials

A semi-structured interview schedule was used as a guide. This schedule probed women's attitudes towards cancer in general and anticipated reactions to a cancer symptom (reported elsewhere^17^), before discussing their experiences of cervical screening and barriers to attending. Women were asked to describe their initial thoughts when receiving a screening invitation and their thought processes around attending or not attending. When this discussion was exhausted, women were encouraged to discuss what they felt would be barriers to attending cervical screening for others. Women were asked open questions and encouraged to give in-depth descriptions. Following the interview, a short demographic questionnaire was completed assessing age, marital status, ethnicity, religion, birth place and screening history.

### Analyses

After familiarisation with the data, a conceptual framework was developed, data were organised using the ‘framework’ feature in Nvivo 9™ (QSR International, Daresbury, UK). Framework is a “matrix-based analytic method which facilitates rigorous and transparent data management” (p.220).^18^ Analysis involved close inspection of each theme, drawing on commonly mentioned attitudes and experiences. Close attention was paid to whether themes arose exclusively or more commonly among ethnic minority women. We have used the terms ‘Asian’, ‘Black’ and ‘ethnic minority’ in the results where appropriate. Definitions of how we used these terms are given in Table 1.

**Table 1:** Definition of terms and how these have been used in the results section

**Term**	**Definition**
White British	Women from *White English/Welsh/Scottish/Northern Irish/British* backgrounds.
Ethnic minority	Women from any ethnic group other than *White English/Welsh/Scottish/Northern Irish/British*. Used in the results section when themes were mentioned by women from several of the ethnic minority groups, but not mentioned by *White British* women.
Asian	Women from *Indian, Pakistani* or *Bangladeshi* backgrounds. Used in the results section when themes were mentioned by women from two or more of these subgroups.
Black	Women from *Caribbean/mixed White & Black Caribbean/African or other Black backgrounds*. Used in the results section when themes were mentioned by women from two or more of these subgroups.
Indian; Pakistani; Bangladeshi; Caribbean; White & Black Caribbean; African; other Black; White other	Ethnic minority subgroups used in the results section when themes were mentioned exclusively by women in one of these subgroups and used in the participant identifier information following each illustrative quote.

## Results

We conducted 43 interviews with women from ethnic minority backgrounds and 11 with women from White British backgrounds (Table 2). Mean age was 46 (range 28–63) years, and women were mostly married/cohabiting (*n*=28) or single (*n*=20). Most reported attending cervical screening regularly, but six women reported not currently being up-to-date with screening, eight had missed or delayed a screening test in the past, and one woman said she had never been screened (Table 3).

**Table 2:** Ethnicity, religion and birthplace of study participants

**Characteristic**	*n*
Ethnicity	
Indian	24
Pakistani	2
Bangladeshi	2
Caribbean/mixed White & Black Caribbean	6
African	2
Black other	4
White other	3
White British	11
Religion	
No religion	11
Christian	14
Hindu	17
Sikh	5
Other (including Muslim, Buddhist, Jewish, other)	7
Place of birth*	
Born in UK and parents born in UK	9
Born in UK and at least one parent born outside the UK	15
Neither self nor parents born in UK	27

*Missing data for three women.

**Table 3:** Cervical screening status

**Status**	White British women (*n*=11) (*n*)	Ethnic minority women* (*n*=43) (*n*)
I have regular smear tests on the NHS	9	26
I have had at least one smear test in the past, but it's more than 3/5 years since my last test	0	6
I have had a smear test recently but in the past there were times when I missed or delayed a smear test	1	7
I have regular smear tests but not in the UK	0	1
I've never had a smear test	0	1
I don't need to have smear tests because I've had a hysterectomy	1	0
Other/missing	0	1

*Missing data for one woman.

NHS, National Health Service.

Five themes were identified in the analyses: (1) lack of knowledge or misunderstanding, (2) the procedure, (3) emotional barriers, (4) practical barriers and (5) cognitive barriers. Each of these themes have been described below with illustrative quotes. Details in parentheses following the quotes represent the participant's identification number (P), ethnicity and age (in years).

### Lack of knowledge or misunderstanding

While many women across all ethnic groups were well-informed about cervical cancer, there were a number of misunderstandings raised in the ethnic minority sample. For example, two women inaccurately described cervical cancer as cancer of other gynaecological sites, for example: “I don't really know much about cervical cancer, err, except yes, it is the cancer that, err is common with women in the ovaries” (P3, Indian, 54). Other Asian women (who were aware of cervical cancer) described a general lack of understanding about cervical cancer within their ethnic group, particularly among older women, or those who have recently migrated, for example: “I think women are not that aware, especially women from my country who come here, what the cervix is even, what cervical cancer is even” (P31, Pakistani, 29).

There were also misperceptions about the causes of cervical cancer and the causal mechanisms involved, for example: “…[women should] use more condoms … that could be a way of protection, because the sperm all the time could cause it, so I've heard anyway” (P18, Black Caribbean, 34). Two women described how they believed sexual abuse caused cervical cancer acting through a psychosomatic pathway, for example: “I know, a friend of a friend was sexually abused, and is currently dying of colon and cervical cancer, and she never dealt with the sexual abuse, and I think the body, experiences emotions… you stuff emotions into the body, and I think, I think that is one level of cause of cancer” (P25, white South African, 41). One woman, having heard about HPV vaccination, had reassessed her perception of cervical cancer risk: “Because I'm not married I always used to think no I shouldn't be worried, but then knowing that if the kids as young as 12 and 13 is suffering that, um, it makes you wonder” (P3, Indian, 54).

When asked about cervical screening, several Asian women who said they had not been for cervical screening or a smear test went on to describe having a smear or a ‘swab’ being taken. Some thought this test was for STIs or was a general health check; others did not know what it was for. In addition, two women (one Indian, one Black Caribbean) described having had cervical cancer, but it became clear through their description that the experience they had was of abnormal cytology: “… [I] became ill along the way … cervical cancer, … I was advised to stop working … it was only after the third test they said you've got abnormal cells … so by the time I went for the colposcopy, um, they were surprised to see that the cells had cleared to my great relief, the cells had cleared I didn't need the colposcopy” (P10, Black Caribbean, 57).

### The procedure

#### The health professional

Many of the women across all ethnic groups seemed to remember clearly the health professionals who had taken their samples: “she was there from when I was little so I kinda grew up with her… so when I went there she made me feel, I always just felt totally relaxed” (P54, Black Caribbean, 37). Bad experiences of screening were often attributed to the health-professional taking the sample: “It just depends on who you have ... she really did put me off going again” (P50, Indian, 43). A few women described the aspects of the patient communication that they felt were important: “I think if they're quite friendly and they relax you, it doesn't make it so uncomfortable … if they could take a bit more time and you know, understand, sometimes they don't have any patience or they just want you in and out” (P18, Caribbean, 34). Many of the women across all backgrounds raised the importance of having the sample taken by a female. Some said they would feel uncomfortable with a male health professional taking the sample; others (mostly Asian women) said they would not attend if the option for a female was not there.

#### Location

Several of the younger women discussed their preferences for cervical screening to be at well woman clinics. These were considered ‘more convenient’ and ‘more anonymous’ than visiting a general practitioner (GP): “it's that reassurity where it's gonna be confidential … cos you know that's where all the people in your community would kind of end up, at the same surgery” (P35, Indian, 30).

### Emotional barriers

#### Fear of pain

Cervical screening was frequently described as painful and uncomfortable, particularly the first time. This issue was raised across all ages and ethnic backgrounds. This meant cervical screening was not considered to be something women wanted to do, although for many this was outweighed by the benefits of screening, for example: “painfully uncomfortable ... but it has to be done, I wouldn't miss one because of that” (P26, White, 52). For some women (all from ethnic minority backgrounds), fear of the test being painful had acted as a barrier to screening in the past: “it's a bit painful, so it's one of the reasons that I have put off and not gone” (P33, Indian, 52).

#### Embarrassment

Embarrassment was also discussed by several women across all ethnic backgrounds. Women described feeling temporarily embarrassed. Several of the women from Black and Indian backgrounds thought this would be a barrier to screening among others, particularly the older generation who would feel that their bodies were private, for example: “I chat with my friends they all would say oh I just don't wanna go you know, I just don't like the whole feeling of somebody doing this and that” (P50, Indian, 43). One woman said this was a barrier for her personally: “I don't want anybody else to look at my own body or touch my own body, that's my own, you know that's my own personal thing so, that could be one of the reasons that's putting me off” (P9, Indian, 45).

#### Fear of cancer

Several women across each ethnic group described fear of cancer or worry about the results of the test as something that had put them off attending screening: “[I've] seen somebody die of cancer it's not nice, so it's quite easy to put your head in the sand” (P35, White, 43). Conversely, reassurance that nothing is wrong was described as a reason for attending promptly: “I'd rather go for peace of mind” (P32, Indian, 38).

#### Shame

A few of the ethnic minority women talked about potential shame if diagnosed with cervical cancer as a barrier to attending screening among others, particularly the older generation: “they are worried about what will people say, why did I get it, how did I get it, questions will be raised and I will have to feel ashamed” (P6, Indian, 52).

### Practical barriers

Several women described cervical screening as an inconvenience: “I think it's just inconvenient, but it's something that we have to do like paying a bill you know the tax council tax is inconvenient” (P54, Black Caribbean, 37). Women felt they had other priorities: “It wasn't that I didn't want to do it, um, I felt that it wasn't a great priority for me at that time, everything else was more important” (P24, Bangladeshi, 48); “I tend to get the letter through and sometimes I get busy so I don't tend to go” (P55, Black Caribbean, 48). This barrier was raised by women across all ethnic groups but was limited to women aged under 40 years. Restricted opening times at surgeries or pre-allocated appointments were described as making attendance particularly difficult for some women. Lack of time to attend and being too busy were common explanations given across all ethnic groups: “putting off going is usually around sort of um, time, you know making time to go” (P26, White, 52). This was largely influenced by work and childcare commitments, for example: “I didn't get round to go to the doctors ... I'm busy cos I'm working full-time ... single parent, lots to do” (P46, White, 44).

### Cognitive barriers

#### Perceived risk

Some women did not feel concerned about cervical cancer and as a result had delayed screening attendance in the past. These women felt cancer would not affect them: “I remember leaving it once because I didn't take it seriously … I just thought oh I'll leave it for a while” (P50, Indian, 43); “it just never seemed hugely important ... I was never afraid that it could be something that could happen to me” (P37, African, 39). Some Asian women justified these feelings of low risk based on their relationship status or sexual behaviour: “I don't need it actually, because I don't have a partner or not married” (P22, Indian, 46). Other women described their concern about cancer as high and therefore felt it necessary to attend for screening. Concerns were influenced by previous family experience and several women also discussed Jade Goody (a celebrity who died of cervical cancer in 2009): “I very quickly went and got myself tested afterwards … it was just something I was putting off, until I got this sort of catalyst” (P20, White, 30).

#### Absence of symptoms

The asymptomatic nature of cervical cancer meant some women felt screening offers a unique opportunity to see if anything is wrong: “if something's going on down there you would never know” (P29, Caribbean, 50). Conversely, the fact that screening involves looking for disease in the absence of symptoms was seen as a reason not to be screened by other women: “It's not pressing because you don't have any symptoms” (P47, White, 37).

## Discussion

The present study explored barriers towards cervical screening, among ethnic minority and White women living in London. Few barriers were exclusively raised by ethnic minority women and most of these were similar to previous qualitative studies with women generally^7–9^ and in specific subgroups.^10–14^ In contrast, language, which has been discussed as a barrier to screening among ethnic minorities in other studies,^10–12^ ^14^ was not a common theme, probably because most of the women we interviewed spoke English.

Ethnic minority women discussed a general lack of knowledge about cervical cancer in their community, particularly among older women. This is consistent with previous qualitative studies^10^ ^12^ ^13^ and quantitative studies that suggest lower awareness of cancer risk factors, warning signs, and screening programmes among ethnic minorities.^19–21^ Several ethnic minority women did not recognise the terms ‘cervical screening’ or ‘smear’, suggesting that the terminology used is not always familiar, even among English-speakers. One study suggested using the term ‘cancer test’,^11^ and while this may not be the most appropriate term (given that cervical screening looks for precancerous changes), it is clear that clarification of cervical screening is an important adjunct to information for ethnic minority women, as well as questionnaires assessing self-reported uptake.

Interestingly, although emotional responses to the procedure (fear, embarrassment, pain) were raised by many women, the potential for these emotions to be barriers seemed more prominent among Asian women. While this needs to be confirmed quantitatively in the screening context, there is already some evidence that Asian women are more likely than White women to report emotional barriers to delayed help-seeking for cancer symptoms.^22^ Some of the ethnic minority women also raised the potential for feelings of shame, if diagnosed with cervical cancer, as a barrier to screening for others in their community. Anticipated shame has been suggested as a barrier to cervical screening in qualitative work with African women^23^ and stigma of cancer has been described among UK ethnic minorities.^24^ ^25^ If perceptions of stigma and shame are barriers to screening, it will be important to address this.

The health professional taking the sample plays an important role in women's perceptions of screening; while the procedure may only be short, women's experiences with the health professional taking the sample and communication during the procedure were well-remembered and for some women this influenced future behaviour. Future research should explore health professionals’ views about screening ethnic minority women. Ensuring smear-takers are culturally competent could help to limit negative experiences at cervical screening among ethnic minority women. Some of the younger women mentioned their preference for having cervical screening at well woman clinics instead of their GP surgery, as this would reduce the chance of seeing other members of their community. Publicising the availability of well woman clinics as an alternative option (when this is available) could help encourage screening attendance for those who have concerns about anonymity.

This study explored barriers to cervical screening in women from a range of ethnic backgrounds. Previous studies have focused on White British/European women^7–9^ or very specific ethnic minority groups (e.g. Somali women).^10^ We tried to ensure that we reached a range of women by recruiting in the community and offering women the opportunity to participate in another language; however, most of the women we interviewed spoke fluent English and had lived in the UK for a long time. This is not surprising given the age range invited to take part, however it means we cannot rule out the possibility of different themes among recent migrants or those who do not speak English. Many ethnic groups were under-represented, which means caution should be taken with extrapolating the findings to all ethnic minority populations.

Women were not identified as non-attenders at the outset and so most had attended cervical screening previously; however, one-quarter of the women were overdue for screening or had delayed screening in the past. The NHS cervical screening programme suggests that around 9% of women (aged 25–64 years) have never been screened; however, only one woman in our sample had never attended cervical screening. The findings here are likely to represent women who intend to undergo screening but delay, rather than those who have made a decision not to attend and have never been screened.^9^ We used women's self-reported screening status and this could be different from their actual attendance. During the interviews women discussed barriers to cervical screening for themselves and others. While it is difficult to determine the validity of these suggested barriers, we felt it was beneficial to gain additional perspectives on barriers to screening from women in ethnic minority communities.

Interventions to improve knowledge and understanding of cervical cancer and the purpose of screening could be of benefit to ethnic minority women. A review of interventions to improve cervical and breast screening uptake in British South Asians found mixed results, with no clear findings about what aspects of interventions are the most effective.^26^ The present study suggests that misunderstandings about the causes of cervical cancer should be addressed and appropriate terminology should be used. Educational interventions offered through social settings, such as community and church groups, may be a good way to encourage discussion about screening among older ethnic minority women. These interventions should focus on emphasising the efficacy of screening and addressing concerns about embarrassment and shame. The continued option of a female health professional is imperative to ethnic minority women, and the opportunity to be screened somewhere other than a GP surgery may also be valuable to those in the younger age groups. Investment in training for health professionals to ensure good communication and empathy may influence repeat attendance for women of all ethnicities.
